# Influence of Pyrolysis Temperature on Cadmium Removal Capacity and Mechanism by Maize Straw and Platanus Leaves Biochars

**DOI:** 10.3390/ijerph16050845

**Published:** 2019-03-08

**Authors:** Haixia Wang, Mingliang Zhang, Qi Lv

**Affiliations:** 1 School of Water Conservancy and Environment, University of Jinan, Jinan 250022, China; stu_wanghx@ujn.edu.cn (H.W.); lvqi7070@163.com (Q.L.); 2 Shandong Provincial Engineering Technology Research Center for Ecological Carbon Sink and Capture Utilization, Jinan 250022, China

**Keywords:** biochar, adsorption, mechanism, cadmium, cation exchange

## Abstract

The influence of pyrolysis temperature on cadmium (Cd) removal capacity and mechanisms by maize straw biochars (MSB) and Platanus leaves biochars (PLB) pyrolyzed at 300, 400, 500 and 600 °C was investigated. The results showed that the biochars pyrolyzed at 500 °C had the highest adsorption capacity for Cd, and the maximum adsorption at pH 5.0 was 35.46 mg/g and 25.45 mg/g for MSB and PLB, respectively. The increase in adsorption efficiency with increasing temperature indicated that the adsorption of Cd onto the biochars was endothermic. Based on the balance analysis between cations (Ca^2+^ and Mg^2+^) released and Cd adsorbed onto biochar in combination with SEM-EDX, FTIR, and XRD analysis, it was concluded that cation exchange, complexation with surface functional groups, precipitation with minerals (CdCO_3_), and coordination with π electrons were the dominant mechanisms responsible for Cd adsorption by MSB. With the pyrolysis temperature increasing from 300 to 600 °C, the contribution of cation exchange (Ca^2+^ and Mg^2+^) on Cd removal by MSB decreased from 37.4% to 11.7%, while the contribution of precipitation with Otavite (CdCO_3_) and Cd^2+^-π electrons interaction increased. For PLB, the insoluble Cd minerals were not detected by XRD, and the contribution of cation exchange had no significant difference for PLB pyrolyzed at 300, 400, 500 and 600 °C.

## 1. Introduction

Cadmium (Cd) is a highly toxic heavy metal with strong mobility, bioavailability and accumulation, which can cause severe damage to human health. Less than 0.005 mg/L cadmium for drinking water has been required by the US Environmental Protection Agency (EPA) standards and Chinese standards for drinking water quality (GB5749-2006). Many methods including chemical precipitation, ion exchange, adsorption and membrane separation have been developed to remove cadmium from wastewater. In recent years, many adsorbents have been successfully used for the removal of Cd from wastewater, including commercial activated carbon, clay minerals, biomass and agricultural waste [1]. Adsorption by using low-cost materials is considered as one of the most efficient methods to remove cadmium from wastewater due to its high efficiency, low cost and environmentally friendly nature [2].

Biochar, a carbon-rich material, has been successfully used for carbon sink, climate change mitigation and soil remediation [3,4,5]. In recent years, biochar has been successfully used to remove heavy metal from wastewater, including Cd [6,7,8]. The adsorption capacity and mechanisms of heavy metal adsorption by biochar could be influenced by the properties of biochar, such as surface area, surface functional groups, cation exchange capacity, and other surface characteristics [9]. The properties of biochars are not only strongly dependent on biomass feedstocks but also on pyrolysis temperature [4,10]. Depending on the biochar type and pyrolysis temperature, the removal of Cd could be attributed to the following mechanisms: precipitation with minerals (e.g., carbonate and phosphate), surface complexation (e.g., –OH, C=O, C–O), cation exchange (e.g., Ca^2+^, Mg^2+^, K^+^ and Na^+^) and coordination with π electrons [4,11]. Chen et al. (2014) prepared municipal sludge biochar pyrolyzed under different temperatures (500 to 900 °C), and investigated the influence of pyrolysis temperature on properties and heavy metal adsorption potential [9]. Xu et al. (2013) studied the efficient removal of Cd from aqueous solutions by the dairy-manure-derived biochar and the maximum sorption capacity was 51.4 mg/g [12]. However, there are relatively few studies focusing on the comparison of adsorption mechanisms for biochars produced from different biomass feedstocks and pyrolysis temperature. The exact removal mechanism of Cd has not been fully understood until now, and the relationship between Cd removal and biochar properties under different preparation conditions is still unclear. Therefore, the mechanisms of Cd removal by biochar pyrolyzed at different pyrolysis temperatures (especially for ion exchange and mineral precipitation) need to be further investigated. In this study, maize straw and Platanus leaves as two feedstocks were used to prepare biochars using a muffle furnace at different pyrolysis temperatures (300, 400, 500 and 600 °C) in a limited-oxygen environment, and then their physio-chemical properties were characterized. The aim of this study was to investigate the effect of pyrolysis temperature on the properties of maize straw and Platanus leaves biochars, and the relationship between pyrolysis temperature and adsorption capacity of Cd; and to reveal mechanisms for Cd adsorption on biochars produced from different feedstocks and pyrolysis temperature.

## 2. Materials and Methods

### 2.1. Preparation of Biochar

Maize straw samples were semi-decomposed in the field on one farm in the Shandong province of China. Platanus leaf samples were collected from the campus of the University of Jinan, China. Maize straw biochar (MSB) and Platanus leaves biochar (PLB) were produced by pyrolyzing the crushed samples (<1 mm) in an oxygen-limited environment at 300, 400, 500 and 600 °C for 2 h in a muffle furnace. After cooling to room temperature under an anoxic environment, the biochars were soaked with deionized water, dried in an oven overnight at 105 °C, and then stored in sealed plastics bag prior to use. The biochars were abbreviated as MSB300, MSB400, MSB500, MSB600, PLB300, PLB400, PLB500, and PLB600 respectively, in accordance with the pyrolysis temperature.

### 2.2. Physical and Chemical Characterization

The elemental composition (C, H, N, S and O) of the biochars was determined using an elemental analyzer (Elementar Vario MACRO cube, Frankfurt, Germany). The pH and electrical conductivity (EC) of the biochars were measured with a pH meter (Leici PHS-3C, Shanghai, China) and a conductivity meter (Leici DDS 307, Shanghai, China) using a biochar-to-water ratio of 1:20 after mixing for 24 h [8]. Considering cation exchange could be one of the potential mechanisms, the contents of water-soluble Ca and Mg from the biochars was measured as follows: 0.2 g biochar was mixed with 25 mL deionized water for 24 h, and the filtrate was used for the measurement of Ca and Mg [9,11,13]. The mineralogical characterization of MSB and PLB was performed by powder X-ray diffraction (XRD) (Bruker D8 Advance, Berliln, Germany). The surface morphology and elemental constitutes of MSB500 and PLB500 before and after Cd adsorption was examined using scanning electron microscope (SEM, FEI Quanta FEG 250, Hillsboro, OR, USA and Hitachi SU 8010, Tokyo, Japan) and energy dispersion spectrometer (EDX). Specific surface area and pore parameters were measured by nitrogen physic-sorption using an ASAP 2020 surface area and porosity analyzer (Micromeritics, Norcross, GA, USA) [11,12,13]. Fourier transform infrared (FTIR) spectra were recorded at wavenumbers in the range of 400–4000 cm^−1^ by the KBr pellet method using an FTIR spectrometer (Nicolet 380, Thermo Scientific, Waltham, MA, USA). 

### 2.3. Batch Adsorption Experiments

#### 2.3.1. Effect of Pyrolytic Temperature on Cd Adsorption

Pyrolysis temperature can affect the physicochemical properties of biochar, which would have a certain impact on Cd adsorption. In order to compare the efficiency of Cd adsorption by the biochars derived from 300, 400, 500, and 600 °C, 0.2 g of biochar sample was added into the plastic bottles containing 25 mL Cd solution (40, 80 and 170 mg/L) at room temperature at pH 4.5–5.0 for 24 h, respectively. 

#### 2.3.2. Adsorption Batch Experiments

A stock solution of 1000 mg/L Cd was made by dissolving Cd(NO_3_)_2_·4H_2_O (analytical reagent grade) in deionized water prepared in a Millipore-Q water system. All solutions were prepared by diluting the stock solution with deionized water for the following adsorption experiments. For adsorption isotherm experiments, 0.2 g of biochar sample was mixed with 25 mL Cd solution with different concentrations at room temperature at pH 4.5–5.0 for 24 h. The concentration of Cd remaining in the filtrate was measured using atomic absorption spectroscopy (AAS) (AA-7000 model spectrometer, Shimadzu, Japan). The adsorbed Cd per unit weight of biochars and the adsorption efficiency of Cd were calculated according to Equations (1) and (2).

(1) $$R(\%)=\frac{C_{0}-C_{t}}{C_{0}}\times 100$$

(2) $$q_{e}=\frac{V(C_{0}-C_{e})}{m}$$

where *q_e_* is adsorption capacity of Cd (mg/g), the *C_0_* and *C_e_* are the initial and equilibrium concentrations of Cd (mg/L), respectively; *V* is the solution volume (L); and *m* is the mass of adsorbent (g).

The effect of solution pH on Cd removal was investigated by mixing the biochars sample (0.2 g) and 25 mL Cd solution (25 mg/L), with an initial pH in the range of 2.0–7.0. Adsorption experiments at higher pH levels can result in the precipitation of Cd(OH)_2_, so the solution pH was controlled to prevent precipitation. In order to reveal the effect of temperature, the 0.2 g sample and 25 mL Cd solution (25, 50 and 90 mg/L) were mixed at different temperatures (20, 30 and 40 °C) (pH 6.0 for MSB500, pH 6.5 for PLB500). The samples were withdrawn at a definite interval of time for the concentration analysis of Cd.

### 2.4. Cation Exchange During Cd Adsorption Process

There were exchangeable cations (Ca^2+^, Mg^2+^, etc.) retained on the biochar, which might have been released into the solution by exchange during the Cd adsorption process [11,12]. The cation exchange mechanism for the adsorption of Cd by biochars was investigated as follows: 0.2 g biochar sample MSB500 or PLB500 was mixed with 25 mL of Cd solution (100, 200, and 400 mg/L) at pH 6.0 for 24 h, respectively. In order to analyze the contribution of cation exchange on Cd adsorption for the biochars pyrolyzed at different temperature, 0.2 g biochar sample (PLB300, PLB400, PLB500, PLB600, MSB300, MSB400, MSB500, and MSB600) was mixed with 25 mL solution (200 mg/L Cd) at pH 5.5 for 24 h, respectively.

The mixtures were filtered through 0.45 μm filter paper for Ca^2+^ and Mg^2+^ concentration analysis. As a control, the release amount of Ca^2+^ and Mg^2+^ after being soaked with deionized water (pH 6.0) was also measured. The amount for net release of Ca^2+^ and Mg^2+^ due to Cd adsorption was calculated by subtracting their amount in control. The experiments were conducted in triplicate.

### 2.5. SEM-EDX, FTIR, and XRD Analysis

The Cd adsorption mechanisms by MSB and PLB were investigated using SEM-EDX, FTIR and XRD. The surface morphology and elemental constitutes of MSB500 and PLB500 before and after Cd adsorption were examined using SEM-EDX. The XRD analysis was conducted to identify the possible formation of crystalline minerals of MSB and PLB before and after Cd adsorption using an X-ray diffractometer. The surface functional groups on biochar before and after Cd adsorption were analyzed by a Nicolet 380 FTIR spectrometer.

## 3. Results and Discussion

### 3.1. Characterization of Biochars

**Figure 1:**
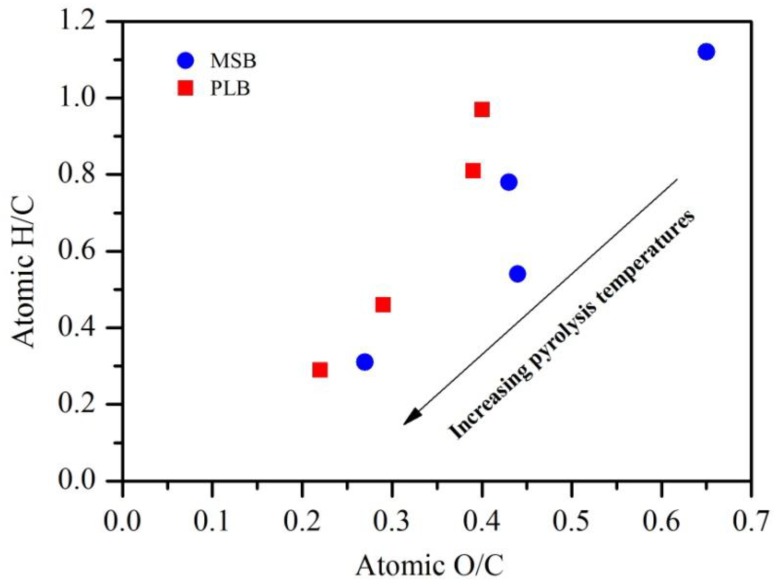
H/C and O/C atomic ratios with increasing pyrolysis temperature.

**Figure 2:**
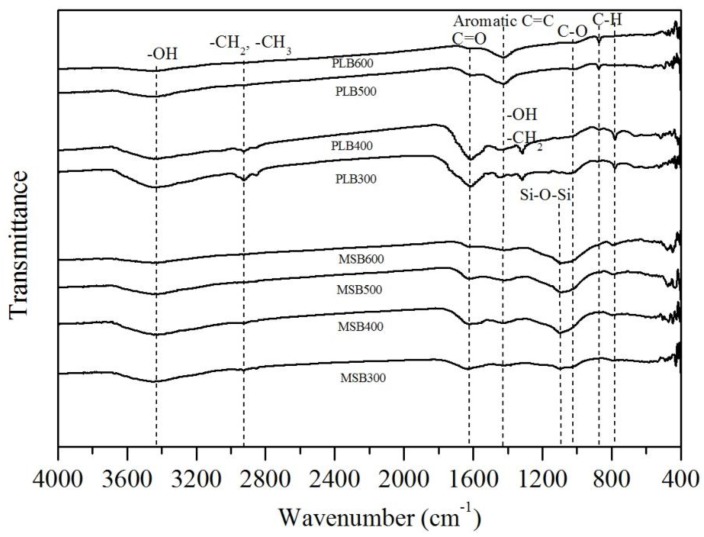
Fourier transform infrared (FTIR) spectra of maize straw biochars (MSB) and Platanus leaves biochars (PLB) pyrolyzed at 300–600 °C.

Pyrolysis temperature affected the physicochemical properties of the biochars. The C content increased with temperature, increasing from 300 °C to 600 °C, while the contents of H and O decreased (Table 1). The decreases in the H/C and O/C atomic ratios with increasing pyrolysis temperature were also observed, indicating carbonization, deoxygenation and dehydrogenation reactions during the pyrolysis process (Figure 1). High pyrolysis temperature promoted the formation of aromatic structure, and the removal of oxygen-containing functional groups (OFGs), which was confirmed by the FTIR analysis (Figure 2). The biochars contain a variety of oxygen-containing surface groups (C=O, C–O, –OH), as well as others (–CH_2_, –CH_3_, aromatic C=C rings, C–H). These peaks of OFGs decreased or disappeared with the increasing pyrolysis temperature. Meanwhile, high pyrolysis temperature promoted the formation of aromatic structure, which was evidenced by the intensified bands at around 781 cm^−1^ (aromatic C–H), 1480 cm^−1^ (aromatic C=C) for MSB, and around 798 cm^−1^ (aromatic C–H), 1384 cm^−1^ (aromatic C=C) for PLB [12].

**Figure 3:**
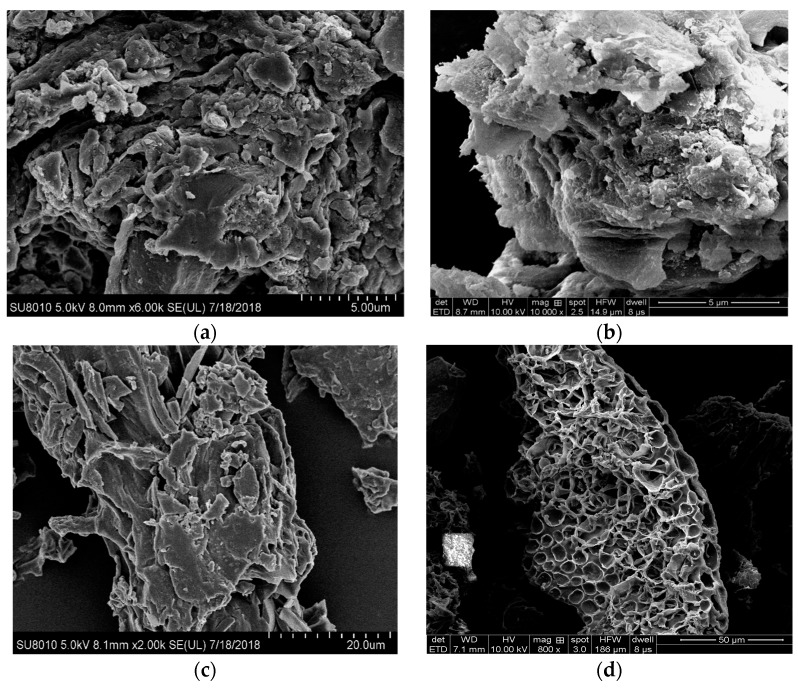
SEM images of (**a**) MSB300, (**b**) MSB500, (**c**) PLB300, and (**d**) PLB500.

As shown in Figure 3, MSB300 and PLB300 demonstrated a rough and compact surface containing very few pores, while MSB500 and PLB500 had a loose and porous structure, which was also confirmed by the larger specific surface area and pore volume for biochars at a high pyrolysis temperature. With pyrolysis temperatures increasing from 300 °C to 600 °C, the specific surface area significantly increased. For example, the specific surface area of MSB increased from 10.3 m^2^/g to 31.1 m^2^/g for MSB, and pore volume increased from 0.034 m^3^/g to 0.062 m^3^/g. The increase in specific surface area was mainly due to the destruction of organic functional groups and the formation of microspores in biochars at high temperature pyrolysis [9].

With pyrolysis temperatures from 300 to 600 °C, the pH of the biochars increased from 7.4 ± 0.2 to 10.1 ± 0.1, from 7.3 ± 0.1 to 9.6 ± 0.1 for MSB and PLB, respectively. It proved a significant positive correlation between pH value and pyrolysis temperature [13]. The EC of PLB increased from 1.35 ± 0.04 mS/cm at 300 °C to 1.64 ± 0.07 mS/cm at 600 °C, while it decreased from 1.75 ± 0.03 mS/cm to 0.76 ± 0.02 mS/cm with pyrolysis temperatures increasing from 300 °C to 600 °C for MSB.

**Figure 4:**
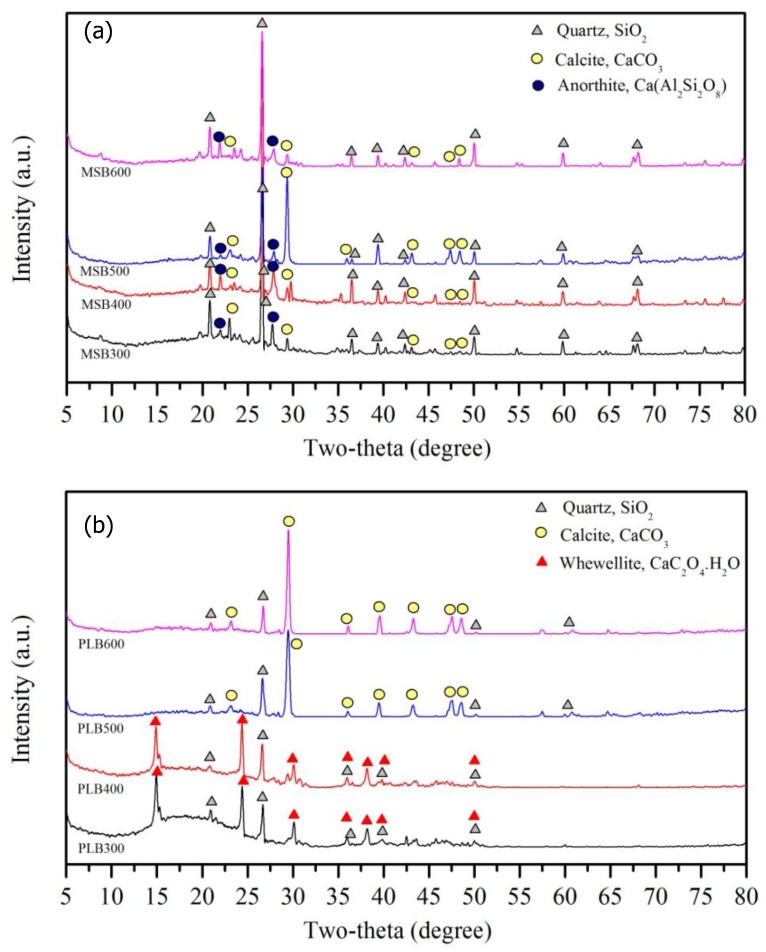
X-ray diffraction (XRD) patterns of MSB (**a**) and PLB (**b**).

**Figure 5:**
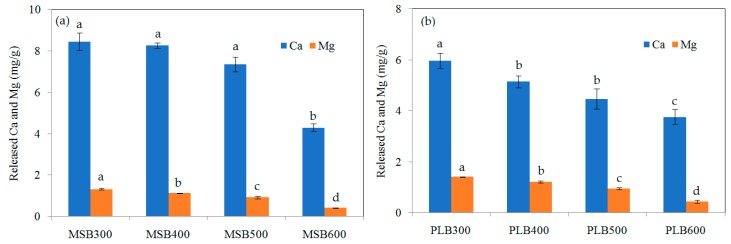
Water-soluble amounts of Ca^2+^ and Mg^2+^ for (**a**) MSB and (**b**) PLB pyrolyzed at 300, 400, 500 and 600 °C at pH 4.5–5.0. The bars marked by different letters indicate significant differences (*p* < 0.05).

As shown in Figure 4, XRD analysis showed that quartz, calcite and anorthite were the main minerals for MSB, and the main minerals of PLB included whewellite, quartz and calcite. The pyrolysis temperature had a significant influence on the minerals’ formation. Whewellite and quartz were the main minerals for PLB300 and PLB400, while calcite and quartz were the main minerals for PLB500 and PLB600 because whewellite was converted to calcite when the pyrolysis temperature increased above 500 °C. For MSB, the amounts of carbonates were substantially influenced by the pyrolysis temperature, and the intensity of the crystal peaks assigned to calcite (CaCO_3_) was the largest for MSB500. The released amounts of Ca and Mg decreased with the pyrolysis temperature increasing from 300 to 600 °C, due to the formation of crystalline minerals at high temperatures (Figure 5). This indicated that pyrolysis temperature could have a significant influence on the adsorption performance as well as the mechanisms.

### 3.2. Comparison of Cd Removal Efficiency at Different Pyrolytic Temperatures

**Figure 6:**
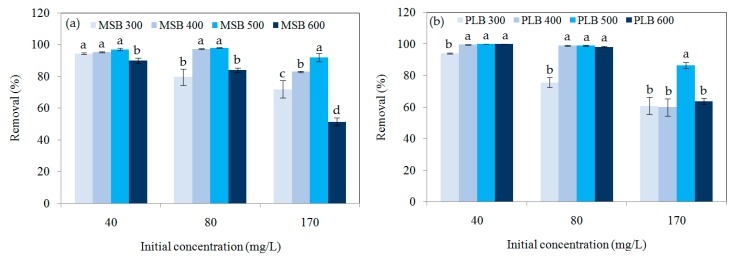
Comparisons of Cd removal by (**a**) MSB and (**b**) PLB from different pyrolytic temperatures.

The removal efficiencies of Cd by MSB and PLB at different pyrolytic temperature are shown in Figure 6. Overall, it decreased with the initial concentration increasing from 40 to 170 mg/L for the biochars. It showed the biochars pyrolyzed at 500 °C had the highest removal efficiency for Cd. For example, the removal efficiency of Cd by MSB300, MSB400, MSB500, and MSB600 was 94.4 ± 0.5%, 95.3 ± 0.3%, 97.1 ± 0.8%, and 90 ± 2% for the initial Cd concentration of 40 mg/L, respectively. It was 80 ± 5%, 97.3 ± 0.2%, 97.9 ± 0.3%, and 81 ± 1% for the initial concentration of 80 mg/L, respectively. It was 72 ± 5%, 83 ± 0.5%, 92 ± 3%, and 51 ± 2% for the initial concentration of 170 mg/L, respectively.

The difference of Cd removal efficiency became more significant with the concentration increasing from 40 to 170 mg/L. One-way ANOVA analysis showed that the removal efficiency of Cd (170 mg/L) by MSB500 was significantly higher than those of MSB300, MSB400 and MSB600 (*p* < 0.05). Similarly, Cd removal by PLB500 was significantly higher than PLB300, PLB400 and PLB600 (*p* < 0.05). The results indicated that pyrolysis temperature affected the capacity of Cd adsorption on the biochars.

### 3.3. Isotherm of Cd Adsorption on Biochars

The Langmuir and Freundlich isotherm models were used for the analysis of adsorption equilibrium data. The linear form of the Langmuir and Freundlich isotherm model can be represented as:

(3) $$\frac{c_{e}}{q_{e}}=\frac{1}{bq_{\max}}+\frac{c_{e}}{q_{\max}}$$

(4) $$\log q_{e}=\log k_{f}+\frac{1}{n}\log c_{e}$$

where *q_e_* is the amount of Cd adsorbed at equilibrium (mg/g), *C_e_* is the equilibrium concentration of Cd (mg/L), *q*_max_ is the maximum adsorption capacity (mg/g), and *b* (L/mol) is the adsorption equilibrium constant. *k_f_* (mg/g) and 1/*n* (unitless) are the Freundlich model constants related to adsorption capacity and adsorption intensity, respectively. 

**Figure 7:**
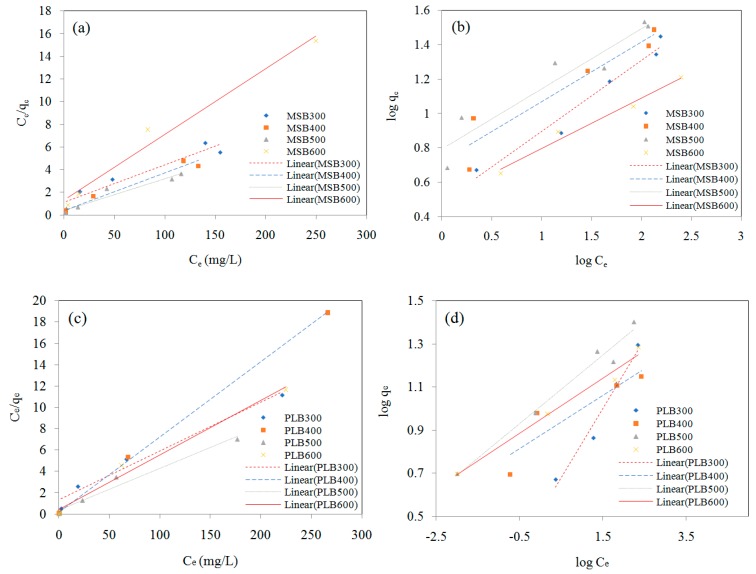
The linear Langmuir ((**a**) (MSB), (**c**) (PLB)) and Freundlich ((**b**) (MSB), (**d**) (PLB)) isotherms of Cd on MSB and PLB from different pyrolytic temperatures at pH 4.5–5.0.

The linear form of Langmuir and Freundlich isotherms of Cd on MSB and PLB from different pyrolytic temperatures are shown in Figure 7. The parameters of Langmuir and Freundlich isotherm models are listed in Table 2. It showed that Cd adsorption onto MSB and PLB fitted both the Langmuir (R^2^ values of 0.93–0.99) and Freudlich model (R^2^ values of 0.82–0.99), implying that chemical and physical adsorption may be involved in Cd removal. The MSB and PLB pyrolyzed at 500 °C had the highest adsorption capacity for Cd, and the maximum adsorption capacities calculated by the Langmuir isotherm were 35.46 mg/g and 25.45 mg/g, respectively. According to the Langmuir model, a dimensionless constant separation factor (R_L_) which can be used to predict whether an adsorption system is favorable or unfavorable [14]. The value of R_L_ indicates the type of isotherm: unfavorable (R_L_ > 1), linear (R_L_ = 1), favorable (0 < R_L_ < 1) or irreversible (R_L_ = 0). The R_L_ values for these biochars were in the range of 0–1, indicating that adsorption of Cd on these biochars was favorable under the experimental conditions and had a high affinity for Cd.

### 3.4. The Effect of pH and Temperature

**Figure 8:**
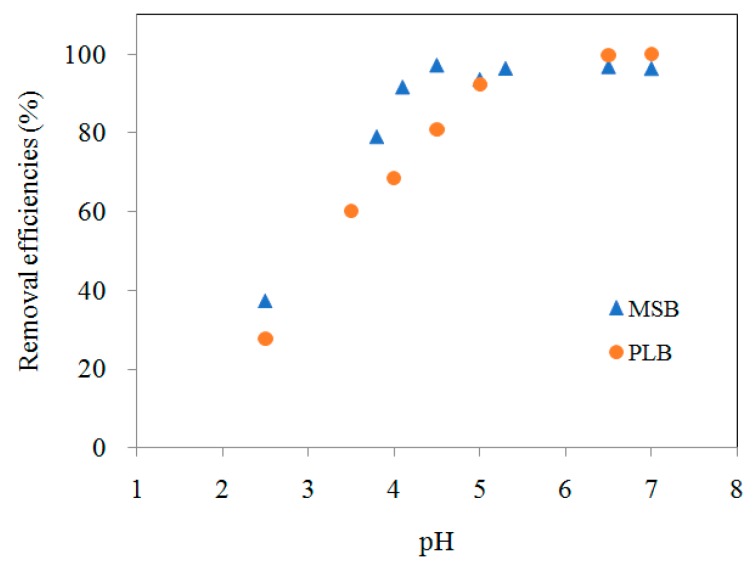
Effect of pH on the adsorption of Cd (25 mg/L).

The pH of a solution is a major factor affecting Cd adsorption from aqueous solutions (Figure 8). The species distribution of Cd is mainly dependent on solution pH, and hydrolyzed metal species (e.g., Cd(OH)^+^, Cd_2_(OH)^3+^) can be formed at a high pH (5.0–7.0) according to the species distribution simulation. A hydroxyl-metal complex has higher adsorption affinity than a hydrated metal ion, as the existence of a hydroxyl group (–OH) can reduce the free energy for adsorption [15]. Besides, at a low pH, the H^+^ concentration is high, which can result in competition with heavy metal ions for surface adsorption sites. Therefore, high pH can increase Cd adsorption efficiency. It should be noticed that adsorption experiments performed at a higher pH (>~6.5) can result in the precipitation of Cd(OH)_2_, which could mislead the adsorption capacity of Cd by biochars due to adsorption. When pH increased from 2.5 to 6.5, the adsorption efficiency of Cd by MSB500 and PLB500 increased from 37.3% to 96.7% and 27.8% to 99.7%, respectively, and the equilibrium was reached (Figure 8). Thus, the optimal solution pH 6.5 was selected for Cd adsorption by the biochars.

**Figure 9:**
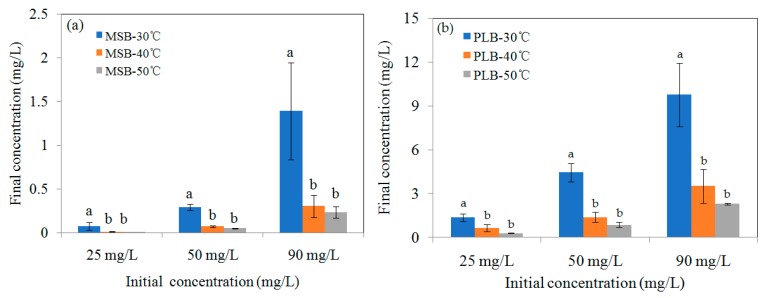
Effect of temperature on adsorption efficiency of Cd by (**a**) MSB500 and (**b**) PLB500 (pH 6.0 for MSB500, pH 6.5 for PLB500).

Increasing temperature can promote the diffusion of Cd^2+^ across the external boundary layer and the internal pores of the biochar particle, owing to the decrease in the viscosity of the solution. In addition, increasing temperature can alter the equilibrium capacity of the adsorbent [16]. The effect of temperature on Cd adsorption onto MSB500 and PLB500 is shown in Figure 9, which showed that the final concentration of Cd decreased with increasing temperature. For example, when the temperature increased from 30 to 50 °C, the final concentration of Cd for MSB500 decreased from 0.07 ± 0.04 mg/L to 0.004 ± 0.001 mg/L for the initial Cd concentration of 25 mg/L, respectively. It was 0.295 ± 0.04 mg/L, 0.07 ± 0.01 mg/L and 0.05 ± 0.01 mg/L for the initial concentration of 50 mg/L, respectively. It was 1.39 ± 0.56 mg/L, 0.30 ± 0.12 mg/L and 0.24 ± 0.07 mg/L for the initial concentration of 90 mg/L, respectively. The difference of Cd removal efficiency became more significant with concentration increasing from 25 to 90 mg/L (*p* < 0.05). The increase of adsorption efficiency with increasing temperature indicated that the adsorption of Cd onto the biochars was endothermic.

### 3.5. Adsorption Mechanism

#### 3.5.1. Cation Exchange

**Figure 10:**
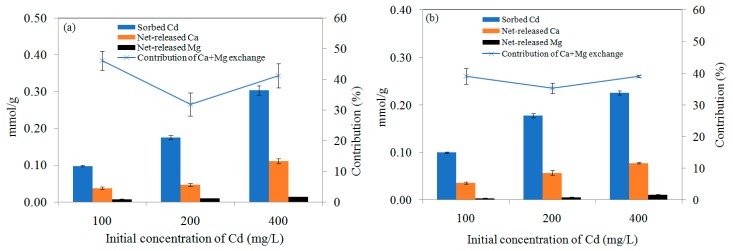
Net-released Ca^2+^ and Mg^2+^ for (**a**) MSB500 and (**b**) PLB500 during Cd adsorption with the initial concentration increasing from 100 to 400 mg/L at pH 6.0.

There are usually many exchangeable metal ions (Ca^2+^, Mg^2+^, K^+^, Na^+^, etc.) retained on the biochar, which could be exchanged by Cd^2+^ in solution during the adsorption process [8,11,12,13]. As shown in Figure 10, an apparent release of Ca^2+^ and Mg^2+^ occurred during Cd^2+^ adsorption on MSB and PLB, and a negligible contribution was observed in the amount of K^+^ and Na^+^ released. The monovalent cations (K^+^, Na^+^, etc.) can form outer-sphere complexation via electrostatic forces, and usually cannot be coordinated with surface functional groups of biochar or form precipitates [11]. While the released divalent alkaline earth cations (Ca^2+^ and Mg^2+^) could release from the complexed surface functional groups (e.g., C=O, C–O, Si–O) and precipitate on the biochar surface [8,11,12,13].

The net-released amount of Ca^2+^ and Mg^2+^ increased with the increasing initial Cd^2+^ concentration (Figure 10). For example, with the initial Cd^2+^ concentration increasing from 100 to 400 mg/L, the adsorbed Cd by MSB500 increased from 10.97 ± 0.20 mg/g (0.098 ± 0.002 mmol/g) to 34.03 ± 1.46 mg/g (0.304 ± 0.013 mmol/g). Meanwhile, the net released amounts of Ca^2+^ and Mg^2+^ increased from 1.513 ± 0.122 mg/g (0.038 ± 0.003 mmol/g) to 4.449 ± 0.295 mg/g (0.111 ± 0.007 mmol/g), 0.178 ± 0.027 mg/g (0.007 ± 0.001 mmol/g) to 0.328 ± 0.006 mg/g (0.014 ± 0.0002 mmol/g), respectively. It suggested cation exchange was responsible for Cd removal by the biochar. The contribution of Ca^2+^ exchange (26.6%–38.6%) on Cd adsorption by MSB500 was significantly larger than Mg^2+^ (4.5%–7.6%).

**Figure 11:**
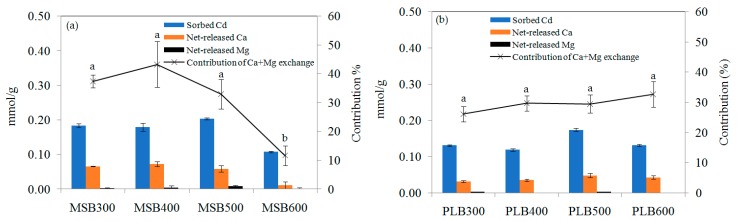
Net-released Ca^2+^ and Mg^2+^ during Cd adsorption for (**a**) MSB and (**b**) PLB pyrolyzed at 300, 400, 500 and 600 °C at pH 5.5.

As shown in Figure 11, for the same initial concentration (200 mg/L), the contribution of cation (Ca^2+^ and Mg^2+^) exchange on Cd^2+^ sorption was 37.4 ± 2.2%, 43.3 ± 7.9%, 32.9 ± 5.2% and 11.7 ± 3.4% for MSB300, MSB400, MSB 500 and MSB600, respectively. This could be related to the formation of crystalline minerals at high temperatures. The contribution of cation (Ca^2+^ and Mg^2+^) exchange to Cd^2+^ adsorption was 26.1 ± 2.5%, 29.7 ± 2.6%, 29.5 ± 3.0% and 32.7 ± 4.2% for PLB300, PLB400, PLB500 and PLB600, respectively. It suggested the contribution of cation exchange on Cd adsorption was more significant for the low-temperature MSB (<500 °C), while there was no significant difference for PLB pyrolyzed at 300, 400, 500 and 600 °C.

#### 3.5.2. Precipitation with Minerals

**Figure 12:**
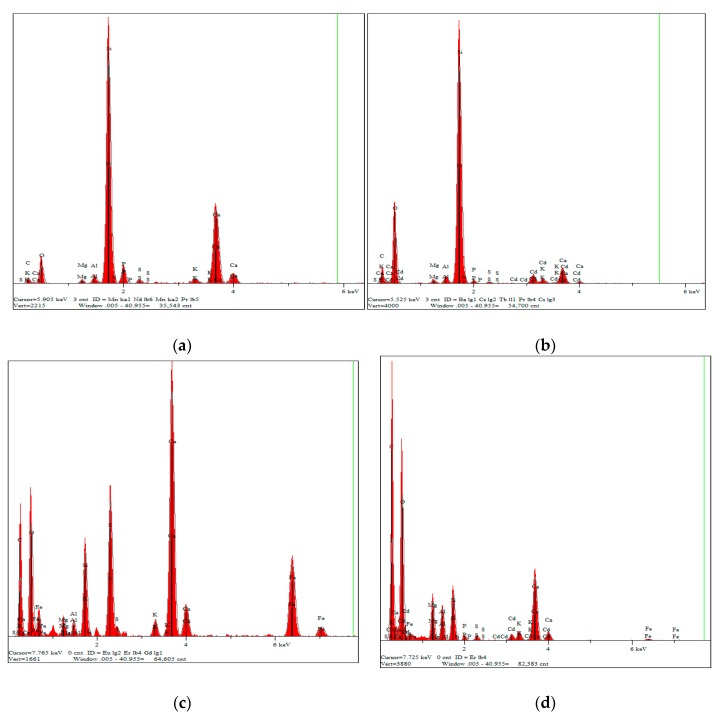
Energy dispersive X-ray (EDS) analysis (**a**) MSB500, (**b**) Cd-loaded MSB500, (**c**) PLB500, and (**d**) Cd-loaded PLB500.

Elemental constitutes of MSB500 and PLB500 before and after Cd adsorption were examined using SEM-EDX (SEM, Quanta FEG 250). As shown by the EDX spectra, Si, C, O, Ca, P, Al, Mg, K, and S were the main elements on the MSB500 surface (Figure 12a). The remarkable peak of Cd appeared for the MSB500 sample after adsorption (Figure 12b). It was shown that C, O, Ca, Fe, Mg, S, K and Ca were the main elements on PLB500 surface (Figure 12c). The remarkable peak of Cd was detected for the PLB500 sample after adsorption (Figure 12d).

**Figure 13:**
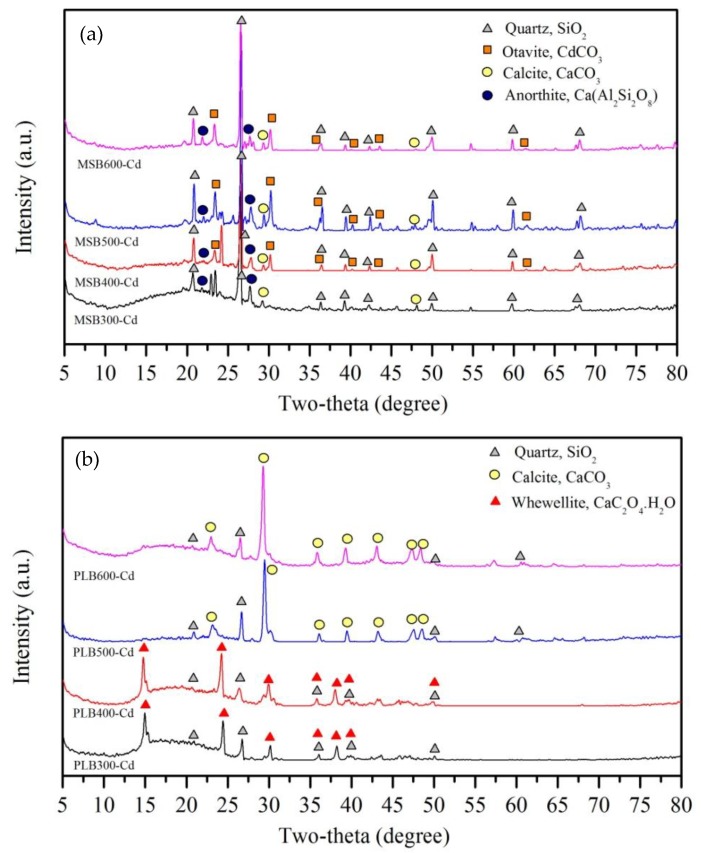
XRD patterns of MSB (**a**) and PLB (**b**) after reaction with Cd.

To confirm the chemical precipitation for Cd adsorption, the original and Cd-loaded MSB and PLB were scanned by XRD, and the spectra are presented in Figure 13. As indicated by XRD, a new peak of CdCO_3_ (Otavite), with the typical 23.45, 30.25, 36.53, 40.25 degree in the 2-Theta, were observed in MSB400, MSB500 and MSB600 after reaction, indicating that CdCO_3_ was the major compound of the precipitate [11]. In addition, the peak intensity of CdCO_3_ (Otavite) was most apparent for MSB500, followed by MSB600 and MSB400. While, no obvious CdCO_3_ (Otavite) mineral precipitate was found in MSB300. It suggested that precipitation with Otavite, (CdCO_3_) was not the main mechanism for MSB pyrolyzed at a low temperature (300 °C). Similar results were reported by another study [10,16]. Precipitation with minerals was not the main removal mechanism for PLB, as the insoluble Cd minerals were not detected by XRD.

#### 3.5.3. Surface Complexation with oxygen-containing functional groups (OFGs) and Coordination with π Electrons

**Figure 14:**
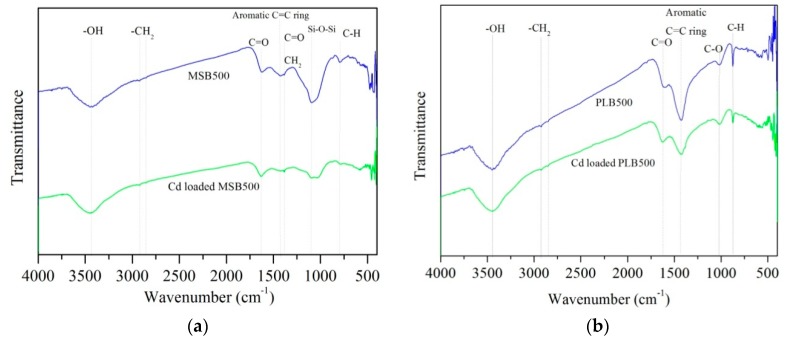
FTIR spectra of MSB500 (**a**) and PLB500 (**b**) before and after Cd adsorption.

Complexation with OFGs has been suggested as one of the major mechanisms for Cd adsorption by biochar [17,18,19,20,21]. After Cd adsorption, the peaks at 1620 cm^−1^ and 1089 cm^−1^ (assigned to C=O and Si–O) of MSB500 weakened, suggesting that OFGs (C=O and Si–O) are involved in the Cd adsorption process (Figure 14).

With the pyrolysis temperature increasing, the peaks at 781cm^−1^ (aromatic C–H) and 1480 cm^−1^ (aromatic C=C structure) for MSB, and 798 cm^−1^ (aromatic C–H), 1384 cm^−1^ (aromatic C=C structure) for PLB became more pronounced (Figure 2), indicating that more pronounced aromatic structure was formed in biochars pyrolyzed at a high temperature. The aromatic structure can provide π-electrons, which have been reported to have the potential to bond heavy metal cations strongly via Cd^2+^-π interaction [11,22]. As shown in Figure 14, after Cd adsorption, the peaks of aromatic C–H and aromatic C=C weakened obviously, and a sharp shift occurred for MSB500 and PLB500. It indicated the participation of Cd^2+^-π interaction in the Cd adsorption process, and the aromatic structure formed in high temperature biochars promoted the Cd^2+^-π interaction [11].

**Figure 15:**
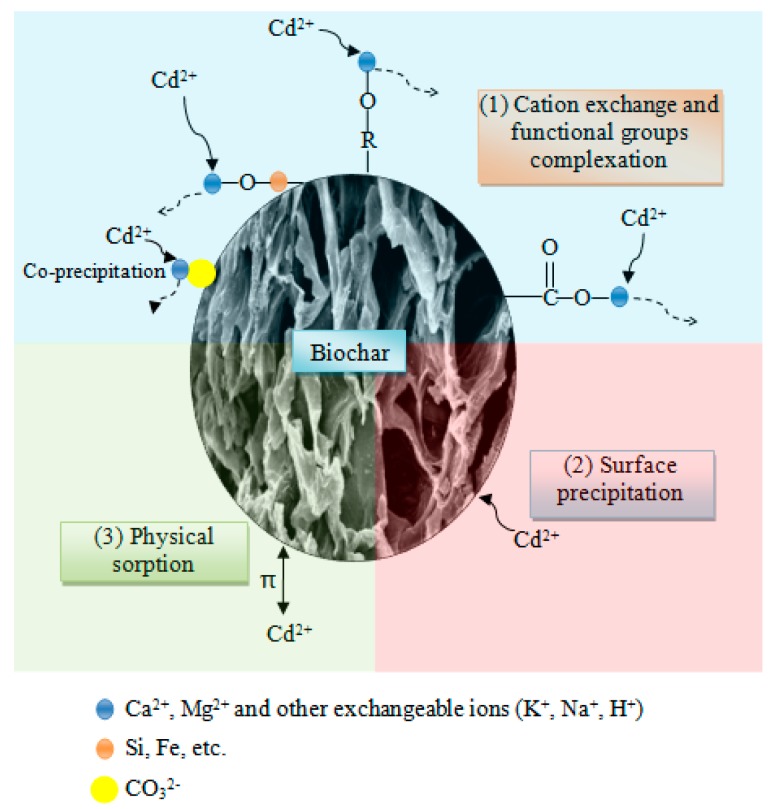
The mechanisms of Cd removal by biochars.

As shown in Figure 15, the proposed mechanisms involved in Cd adsorption by the biochars in this study included: (1) ion exchange and functional groups complexation, (2) precipitation or co-precipitation of Cd as CdCO_3_, (3) coordination with π electrons.

## 4. Conclusions

The results concluded that pyrolysis temperature had a great effect on the capacity and mechanism of Cd adsorption on the biochars. Maize straw biochars (MSB) and Platanus leaves biochars (PLB) pyrolyzed at 500 °C had the highest adsorption capacity for Cd based on the Langmuir model. With pyrolysis temperature increasing from 300 °C to 600 °C, the contribution of cation exchange (Ca^2+^ and Mg^2+^) on Cd removal by MSB decreased, while the contribution of precipitation with Otavite (CdCO_3_) and Cd-π interaction increased. For PLB, the contribution of cation exchange had no significant difference for PLB pyrolyzed at 300–600 °C, and precipitation with minerals was not the main removal mechanism. 

## Figures and Tables

**Table 1:** Physio-chemical properties of MSB and PLB pyrolyzed at 300, 400, 500 and 600 °C.

Biochar	pH	Ec (mS/cm)	Ca * (mg/g)	Mg * (mg/g)	Elemental Composition					Molar Ratio		Surface Area (m^2^/g)	Pore Volume (cm^3^/g)
					C (%)	H (%)	O (%)	N (%)	S (%)	H/C	O/C		
MSB300	7.4 ± 0.2	1.75 ± 0.03	8.46 ± 0.43	1.32 ± 0.05	24.14	2.25	20.93	2.19	0.96	1.12	0.65	10.3	0.034
MSB400	9.1 ± 0.2	1.53 ± 0.02	8.26 ± 0.23	1.13 ± 0.02	25.85	1.68	14.83	1.91	1.16	0.78	0.43	17.1	0.047
MSB500	9.4 ± 0.1	1.10 ± 0.07	7.35 ± 0.35	0.93 ± 0.07	22.08	0.99	12.94	1.41	0.97	0.54	0.44	24.5	0.065
MSB600	10.1 ± 0.1	0.76 ± 0.02	4.30 ± 0.20	0.42 ± 0.02	26.44	0.68	9.54	1.46	0.99	0.31	0.27	31.1	0.062
PLB300	7.3 ± 0.1	1.35 ± 0.04	5.97 ± 0.31	1.41 ± 0.01	52.66	4.25	28.29	1.23	1.05	0.97	0.40	0.2	0.001
PLB400	8.3 ± 0.1	1.06 ± 0.2	5.15 ± 0.24	1.22 ± 0.05	47.56	3.22	24.62	1.19	0.93	0.81	0.39	0.6	0.003
PLB500	9.4 ± 0.1	1.37 ± 0.08	4.47 ± 0.39	0.95 ± 0.04	50.24	1.94	19.45	1.17	0.96	0.46	0.29	7.8	0.023
PLB600	9.6 ± 0.1	1.64 ± 0.07	3.76 ± 0.30	0.44 ± 0.06	54.62	1.32	16.22	1.11	1.03	0.29	0.22	/	/

* Deionized water-soluble.

**Table 2:** The parameters of the Langmuir and Freundlich isotherm models.

Adsorbent	Langmuir			Freundlich		
	*q*_max_ (mg/g)	*b* (L/mg)	R^2^	*k_f_*	1/*n*	R^2^
MSB300	30.30	0.03	0.93	3.03	0.41	0.97
MSB400	30.12	0.09	0.97	5.28	0.35	0.90
MSB500	35.46	0.08	0.94	6.21	0.35	0.90
MSB600	17.21	0.04	0.98	3.19	0.29	0.98
PLB300	21.83	0.03	0.97	3.30	0.32	0.97
PLB400	14.16	0.45	0.99	7.51	0.12	0.82
PLB500	25.45	0.12	0.97	10.20	0.16	0.97
PLB600	19.49	0.13	0.99	8.88	0.13	0.99
